# Fragility fractures of the sacrum: how to identify and when to treat surgically?

**DOI:** 10.1007/s00068-015-0530-z

**Published:** 2015-04-18

**Authors:** D. Wagner, C. Ossendorf, D. Gruszka, A. Hofmann, P. M. Rommens

**Affiliations:** Department of Orthopaedics and Traumatology, University Medical Centre, Johannes Gutenberg-University Mainz, Langenbeckstr. 1, 55131 Mainz, Germany

**Keywords:** Sacrum, Pelvis, Insufficiency fracture, Fragility fracture, Osteoporosis, Treatment

## Abstract

The increasing prevalence of fragility fractures of the sacrum (FFS) occurring predominantly in osteoporotic individuals poses a diagnostic and therapeutic challenge. The clinical presentation varies from longstanding low back pain without the patient remembering a traumatic event to immobilized patients after suffering a low-energy trauma. FFS are often combined with a fracture of the anterior pelvic ring; hence they are classified as a part of fragility fractures of the pelvis (FFP). If not displaced, the patients are treated with weight bearing as tolerated and analgesics; however, we advocate to treat displaced fractures surgically according to the fracture personality and the patient’s comorbidities. Surgical options include minimal invasive sacro-iliac screws, trans-sacral bar osteosynthesis, open reduction and internal fixation, or spinopelvic stabilization. In the light of the high complication rate associated with immobilized patients, an operative approach often is indicated to accelerate the patient’s mobility.

## Introduction

The most common disease weakening the bone in elderly is osteoporosis; thereby primary and secondary types of osteoporosis are distinguished. Primary osteoporosis is found in 70–80 % of the affected individuals including both, postmenopausal and senile osteoporosis. The remaining 20–30 % present with increased bone fragility due to another pathology. Underlying causes are either drugs/pharmaceuticals, such as cortisone or alcohol, endocrinological disorders such as secondary parathyroidism, gastro-intestinal problems, or hematological diseases [1]. Osteoporosis is a disease leading to a general lower bone mass and to an alteration of the bony microarchitecture, thus increasing the risk for pathologic fractures [2].

Epidemiologic changes in first and second world countries will inevitably lead to a constant increase of the elderly population. In the European Union, the number of people older than 50 years will increase by 20 % until 2025, whereas, at the same time, the population of people older than 80 years will increase about 32 % [3]. In the population aged 50 years or older, the prevalence of osteoporosis was reported to be 21 % in women and 6 % in men [4]. In 2010, about 22 million females and 5.6 million males were affected by osteoporosis in the European Union. In 2025, an estimated total of 34 million people will be affected. At the same time, fractures associated with osteoporosis are estimated to increase from 3.5 to 4.5 million per year [3]. As pelvic and sacral fractures in elderly are very likely to be associated to osteoporosis [5] with pelvic fractures making up to 7 % of all osteoporotic fractures [6], an increase of those fractures is to be expected. Currently, an incidence of 92/100,000 persons aged 60 years or more was calculated for pelvic fractures in Finland [7], whereas “only” 25/100,000 were found in Scotland [8]. The incidence of osteoporotic fractures of the pelvis increased from 1970 to 1997 by 460 % [7]; for the time from 2005 to 2025 it is estimated that pelvic fractures in elderly will increase by 56 % [6].

The expected increase in fragility fractures of the pelvis (FFP) and fragility fractures of the sacrum (FFS) poses a significant challenge in orthopedic traumatology. In this review, current literature data on diagnosis, morphology, and classification as well as treatment alternatives are presented.

## Definition—fragility fractures of the sacrum

Fractures as a consequence of a low-energy trauma are often referred as stress, insufficiency, fatigue, or fragility fractures. “Stress” fractures occur after recurrent loading within physiologic ranges; they enclose “fatigue” and “insufficiency” fractures [9]. Fractures due to repetitive stress in healthy bones were classified as “fatigue” fractures. A historically well-known example is a “march fracture” of the metatarsal bones occurring in military recruits [10]. Nowadays, these fractures are seen in recreational and professional athletes. They are typically localized in the proximal tibia, the distal fibula, the metatarsal bones, the navicular bone, or the neck of the femur [11]. Fatigue fractures have been described for the sacrum as well, mostly in young female runners [12]. In contrast, “insufficiency” fractures are caused by a decreased ability of abnormal bone to withstand repetitive, yet sub-threshold stress. However, the classification of “stress” fractures is not conclusive enough dealing with osteoporosis-associated fractures, as they often are caused by a combination of a minor trauma and decreased bone quality and mineralization [13]. Therefore, in a recently published classification of pelvic fractures in elderly, such low-energy fractures in osteoporotic patients were defined as “fragility fractures of the pelvis” (FFP) [13]. This was referred to the definition of the WHO combining the influences of both, the injury type and the reduced bone quality and mineralization [14]. We, therefore, prefer to further use the term “fragility fracture” instead of stress, fatigue or insufficiency fracture to describe osteoporosis-associated fractures due to a minor trauma. To facilitate the review and discussion of past literature, we use the term “FFS” for “sacral insufficiency fractures” (SIF) in elderly.

The main cause of bone fragility in FFS is primary or secondary osteoporosis while only a minority of cases refer to local bone alteration due to radiotherapy or tumor [15]. Pregnancy and lactation leading to secondary osteoporosis were also reported to cause SIF [16]. These fractures are not only attributed to altered bone structure and -mass, but also to biomechanical factors such as hyperlordotic posture, relaxation of pelvic ligaments altering the stability of the pelvic ring and weight gain play an important role [17]. FFS are also observed in patients after undergoing spinal instrumentation. Twenty-four sacral fractures occurred after 394 lumbo-sacral spinal instrumentations extending to L5/S1, corresponding to an incidence of 6.1 %. The fractures were detected after a mean of 4.3 months (2 weeks–21.7 months); a minor trauma was remembered by only 3 patients in that series. The mean age was 67 years, 71 % of patients suffering a fracture were females and the fracture occurred more often in instrumentation involving more than three levels [18]. Another biomechanical stress factor leading to FFS may be degenerative spondylolisthesis on level L5/S1 by increasing the shear forces on the endplate of S1 [19].

## Clinical presentation

Physicians treating elderly patients suffering from low back pain are often unaware of FFS and may not include this entity into their differential diagnosis. As there is only limited information about their incidence, FFS may be underestimated in daily practice. The diagnosis is often delayed as patients are treated for low back pain and appropriate diagnostics may not be used or sacral fractures in elderly were not detected in conventional X-ray. Anecdotic reports even describe cases of wrong surgery (e.g. decompression of the spinal canal) due to low back pain with a fracture of the sacrum being recognized as the major source of pain later [20]. Female patients aged more than 55 years presenting with low back pain were found to have a FFS in 1.8 % when appropriate diagnostics such as computed tomography (CT) or scintigraphy was applied [21]. However, there may be even more patients suffering from FFS, as in 54–98 % of patients presenting with a pubic rami fracture, an additional fracture of the posterior pelvic ring was found as well [13, 22–24].

In the elderly presenting with a FFP, a low-energy trauma, e.g., a simple fall from a standing or even sitting position, is often the only cause remembered. However, such a traumatic event could be found or remembered only in one-third of patients [25], which could be explained by advanced dementia. Even the transfer from bed to a chair [13] or the effort to cough [25] was sufficient to provoke a FFP in some patients. Experimentally, a backward fall from a standing height provokes a force of 3250 ± 600 N [26], which was similar to the force to reproduce a fracture in cadaveric osteoporotic sacrum (3200 ± 1200 N) [27].

Patients often describe a dull pain in their lower back or over the sacrum; in some cases the pain irradiates in a pseudoradicular manner down to their legs. Patients with an additional fracture of the anterior pelvic ring often suffer from pain in their groin [28]. In all elderly patients with lower back, sacrum, or groin pain, a history of trauma has to be elucidated. In patients with a fracture of the pubic rami, the presence of lower back pain was associated with an additional sacral fracture [23].

The physical examination includes careful testing of the stability of the pelvic ring with the patient in supine position. Rotational instability indicates fractures of both, the anterior and posterior parts of the pelvic ring. There may be tenderness over the sacrum itself or over the lower spine. In cases with involvement of the anterior pelvic ring, tenderness in the groin may be present. Physical tests to stress the SI-joint and the sacrum include FABER (flexion-abduction-external rotation test) and Gaenslen’s test [17, 29, 30]; however, they exhibit a low specificity in painful patients.

## Diagnostics

**Fig. 1:**
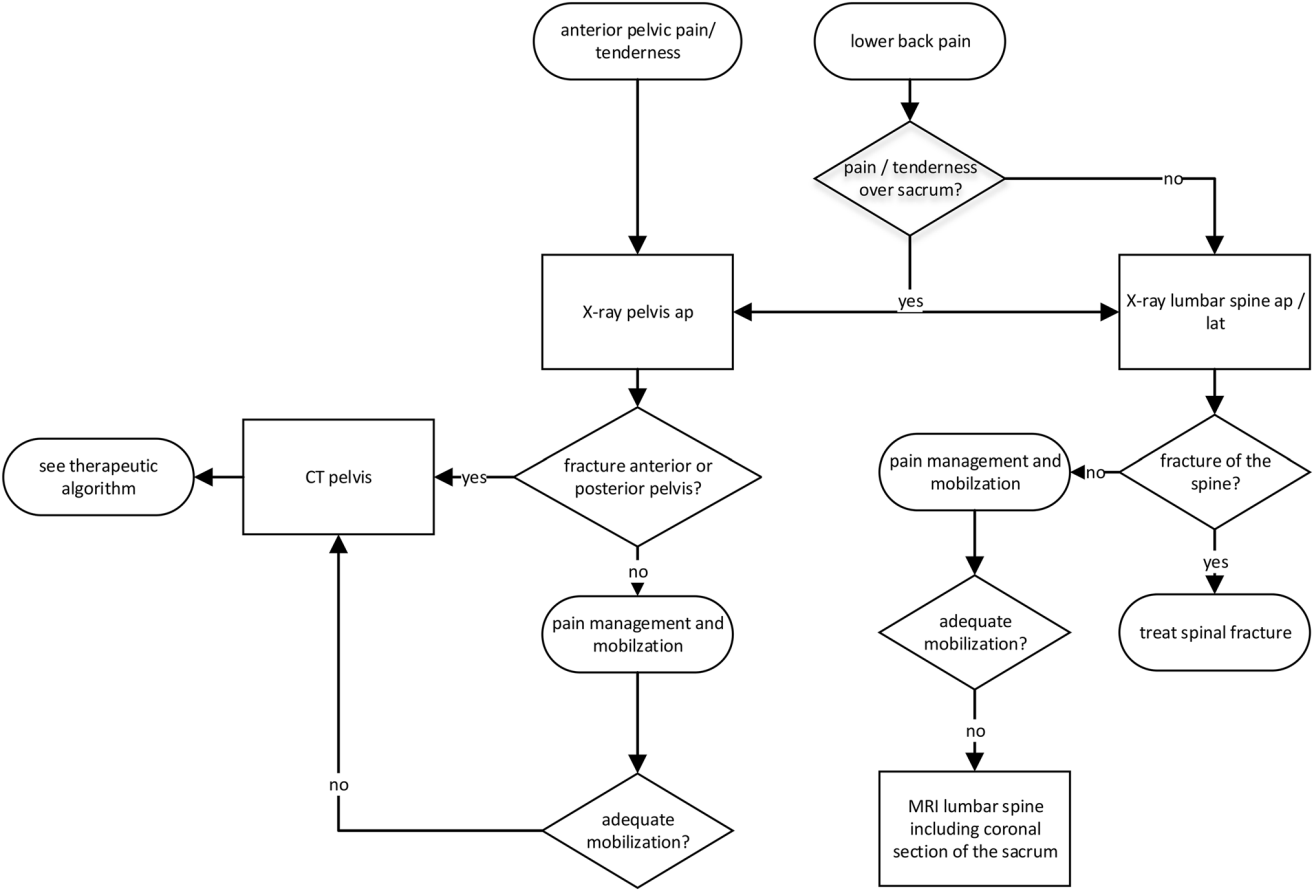
Diagnostic algorithm

The primary diagnostic screening tool in patients with suspected pelvic ring or sacral fractures is an ap-view of the pelvis (Fig. 1). Here, the pelvic ring is inspected for fractures of the pubic rami and the ilium, a diastasis of the symphysis, and cortical irregularities in the posterior pelvic ring. When a fracture of the pubic rami is diagnosed, a CT-scan of the pelvis is performed to assess the full extent of the injury. There, a thorough analysis of cortical irregularities of the sacrum in the axial, sagittal, and coronal reconstructions is compulsory as there may be only discrete signs of a fracture. Inlet and outlet views [31] are mainly required to assess the extent of displacement and instability as well as for preoperative planning of displaced pelvic ring injuries. Conventional X-rays of the lumbar spine are carried out to exclude other pathologies in elderly suffering from low back pain [32].

**Fig. 2:**
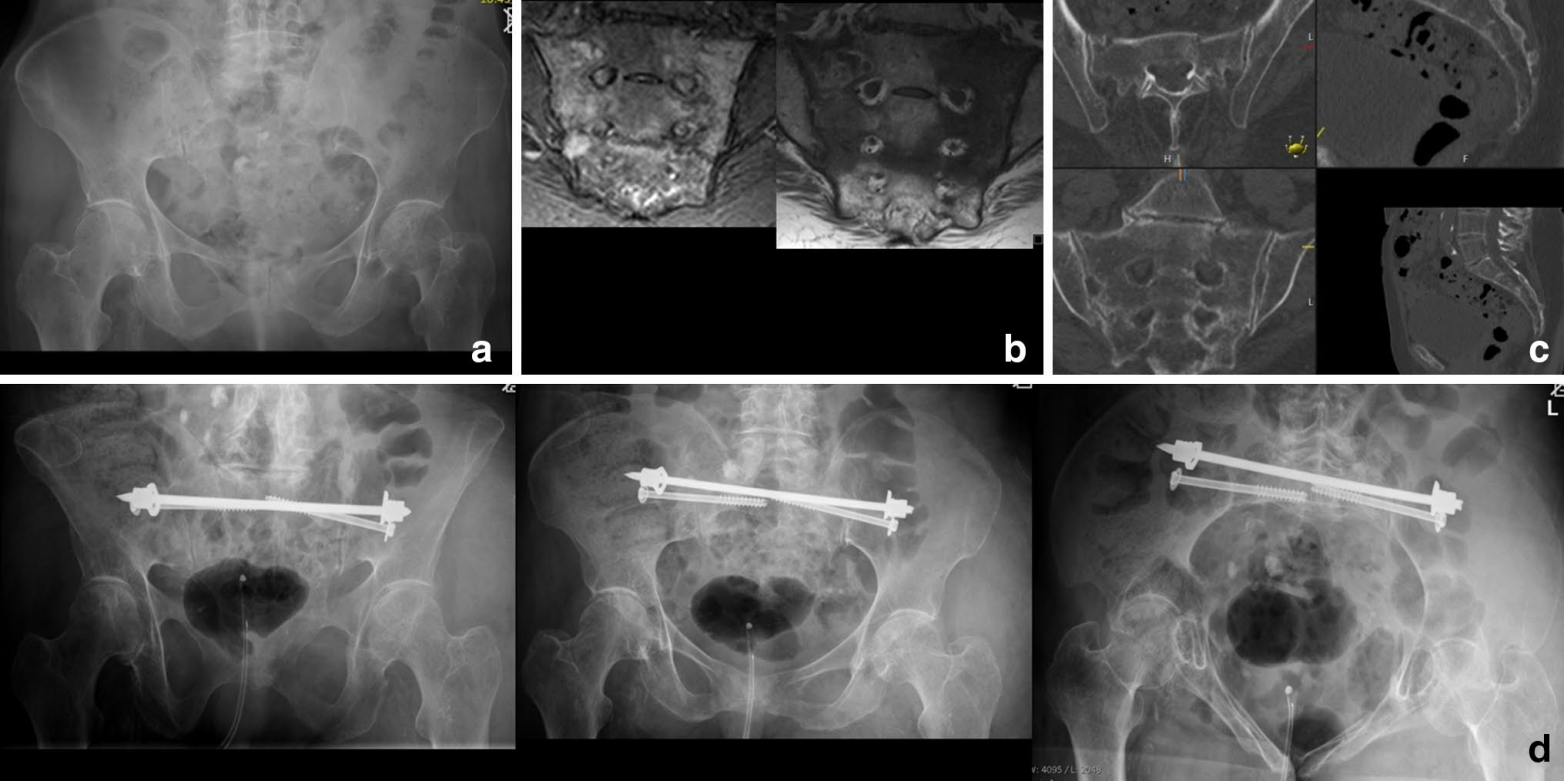
FFP type IIa. 84-year-old female with immobilizing lower back pain. Conventional radiograph did not show a bony lesion (**a**). Also with adequate pain medication mobilization was not possible. The MRI (T1 and STIR sequence in the coronal plane of the sacrum) showed bilateral bone bruise in the sacral ala with a transverse connection on level S2/S3 (**b**). A CT scan confirmed bilateral sacral involvement without fracture of the anterior pelvic ring (**c**). The patient was stabilized percutaneously with a trans-sacral bar and bilateral SI-screws (**d**)

Patients without an evident fracture after completed diagnostics receive analgesics and are mobilized as tolerated. If the lower back or dorsal pelvic pain persists for days, we use a magnetic resonance imaging (MRI) scan of the lumbar spine including coronal oblique images in the plane of the sacrum to exclude occult osteoporotic fractures of the sacrum or the lumbar spine [33] (Fig. 2). We do not recommend the use of scintigraphy anymore.

Fractures of the sacrum are frequently associated with anterior pelvic lesions and vice versa [13, 22–24]. Hence, in a patient with pubic rami fractures, a lesion of the posterior pelvic ring and sacrum is very likely and must be ruled out. However, the diagnosis of sacral fractures using conventional radiographs only is often complicated by overlying bowel and bladder content, or, particularly in the elderly patient, by rarefication of the bone structure, thereby leading to decreased contrast [34]. Using conventional X-ray as primary diagnostic tool, FFS were detected initially in only 0–10 %; retrospectively, a fracture could be detected in 20–34 % of the cases after diagnosing a FFS by other imaging modalities [25, 35–37]. On conventional radiographs, FFS appear as a vertical band of sclerosis in the region of the sacral ala [38], rarely a discontinuation of the cortical bone lateral to the sacral foramina is seen. Particularly in cases an anterior pelvic ring fracture or a spinopelvic dissociation was detected, inlet and outlet pelvic views can be used to assess the extent of dislocation and instability of the pelvic ring. Without recognizing the chronic nature of some FFP, non-united fractures may be mistakenly rated as malignancy and undergo open biopsy [36, 39]. Compared to the low sensitivity of conventional X-ray, CT has a better sensitivity of 60–75 % in detecting FFS [35, 36]. There, FFS often show a discontinuation of the anterior sacral cortex located laterally to the sacral foramina with only minor displacement [40]. Sometimes, a small crush zone medially to the SI-joint can be detected [13]. Occult fractures may not be visible on CT as there is no cortical disruption. However, using MRI, they show a hyper-intense pattern in T2 and STIR (Short Tau Inversion Recovery) sequence called “bone bruise” [41] representing posttraumatic bone hemorrhage. The histological correlate was shown to be microfractures of cancellous bone, edema, and bleeding into fatty bone marrow [42]. MRI has a high sensitivity of 100 % in detecting sacral fractures; however, a fracture line may not be clearly visible in up to 7 % [35]. An adjacent soft tissue edema was detected in 36 % of FFS whereas it was seen in 65 % of pubic rami fractures [35].

Recently, occult fractures of the lumbar spine and the sacrum have been shown to be detectable not only by MRI or scintigraphy but also by multidetector CT. In the sacrum, occult unilateral FFS were detected by measuring the mean Hounsfield Units (HU) in the sacral alae bilaterally at the level of S1, S2, and S3. A cutoff-value of a unilateral increase of 35 HU correlated significantly with the presence of bone bruise in MRI. This is explained by an increase in interstitial fluid due to trabecular bone disruption leading to higher HU [43].

Bone scintigraphy was often referred as diagnostic tool to detect FFS [33]. Typical patterns of uni- or bilateral enhancement in the sacrum and sometimes a “Honda-sign” indicating a H-fracture of the sacrum were present [44]. With MRI being widely available nowadays, scintingraphy is not anymore used to diagnose FFS. A disadvantage of scintigraphy is the lacking possibility to differentiate between a fracture and a metastasis. Further, in MRI it is possible to detect fracture lines as low-intense zones in T1 sequence [35].

## Fracture classification

Sacral fractures are commonly classified according to Denis et al. [45] based on the outcomes of a series of 236 sacral fractures due to high-energy trauma. They defined three zones within the sacrum. Fractures were classified in zone I when being localized in the sacral ala laterally to the sacral foramina, representing 50 % in their series. Zone II fractures were involving the sacral foramina, hence trans-foraminal, constituting 34 % of their fractures. Central fractures, localized in zone III, involved the sacral canal and occurred in 16 %. Roy-Camille [46] further described “suicidal jumper’s fracture” as being a transverse fracture between the vertebral bodies S1 and S2 or at the level of vertebral body S2 combined with bilateral vertical trans-foraminal fracture lines. This leads to a discontinuation of the lumbar spine in relation to the pelvis, functionally resulting in a spinopelvic dissociation and consequently creating a high instability. These classifications are widely used; however, as they were developed for patients suffering from high-energy trauma, they do not represent important characteristics of FFS. In high-energy trauma, the classification of Denis reflects the grade of instability and the risk for neurological impairment [45, 47]. In contrast, in patients suffering a low-energy trauma, instability has different characteristics, usually not leading to severe bleeding or neurological injuries but more often to longstanding and immobilizing pain.

**Fig. 3:**
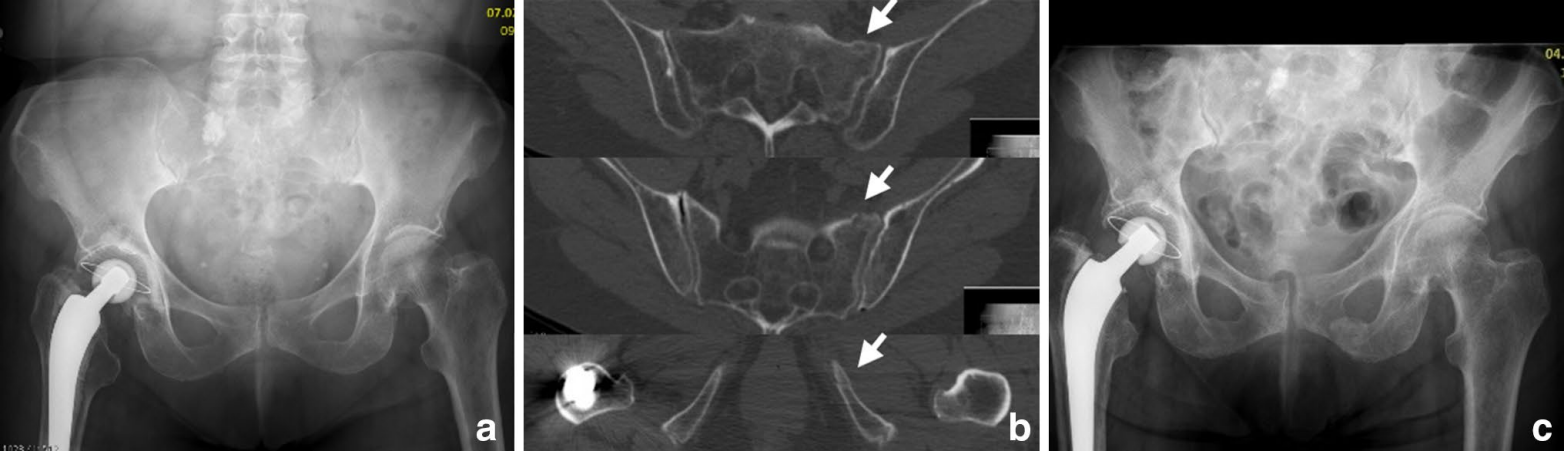
FFP type IIb: 81-year-old female with a crush injury of the left sacral ala and a non-displaced fracture of the left anterior pelvic ring (**b**). With conservative management she went on to consolidation (**c** radiograph 13 months after trauma)

**Fig. 4:**
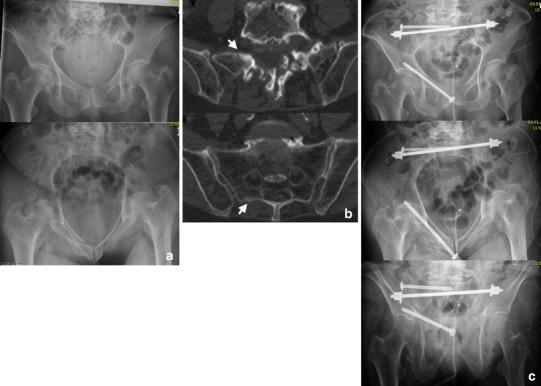
FFP type IIc. 91-year-old female with a unilateral sacral fracture (**b**) and a slightly displaced anterior pelvic ring fracture (**a**). Conservative treatment failed because of persisting pain in the dorsal pelvic ring. Minimal-invasive surgery was performed (**c**): the sacrum was addressed with a trans-sacral bar and a SI-screw on the right side, and the superior pubic ramus was fixed retrogradely with a cannulated screw. Pain at mobilization resided after the operation

**Fig. 5:**
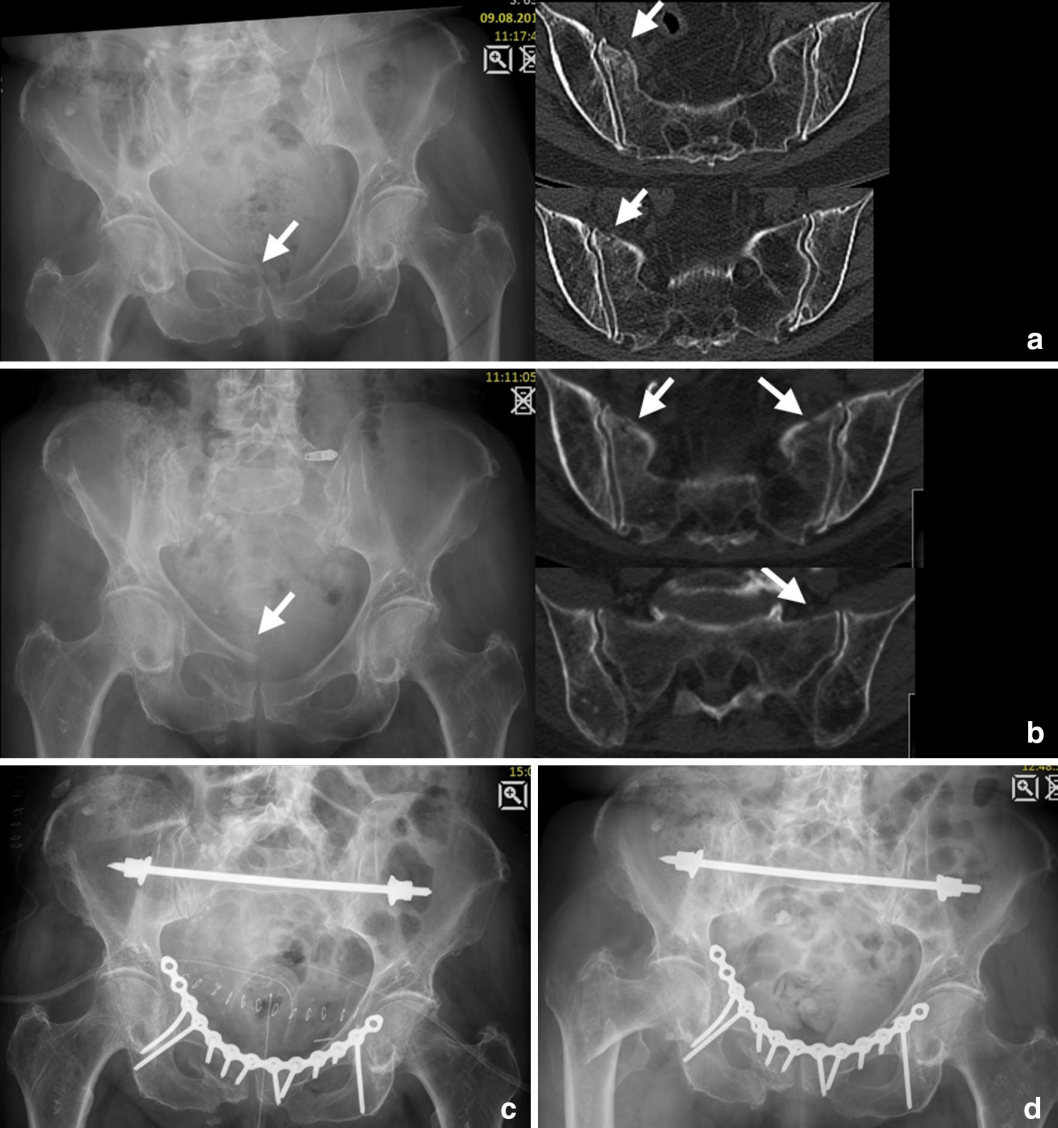
FFP type IIc. Initial diagnostics showed a unilateral fracture of the sacral ala right-sided and a displaced fracture of the anterior pelvic ring in this 83-year-old female (**a**). Conservative treatment with mobilization led to a bilateral sacral fracture and progressive displacement anteriorly after 3 weeks (**b**). She was stabilized subsequently with a trans-sacral bar and an anterior plate osteosynthesis (note the long screws reaching the posterior column) (**c**). A radiograph taken 5 months later demonstrated no implant failure or displacement; however, the patient sustained a pertrochanteric fracture due to recurrent fall (**d**)

**Fig. 6:**
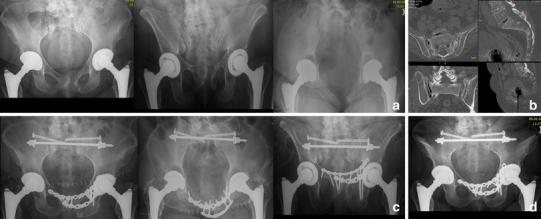
FFP type IVb. This 67-year-old patient presented 10 months after a fall suffering from groin pain and pain projecting in both legs as well as a peroneal lesion on the left side, she was treated conservatively. She had bilateral pseudarthrosis of the sacrum and the left pubic rami with intrusion of the sacrum into the pelvic ring (**a**, **b**). Open debridement was performed in all pseudarthrosis with application of iliac bone graft. The posterior instabilities were addressed with a trans-sacral bar and an additional SI-screw on both sides through S1. Anteriorly, symphysiodesis was performed with bone graft and a double-plate osteosynthesis (**c**). Follow-up at 2 years showed consolidation (**d**). Mobilization was unlimited and without pain

FFS were recently included into a classification of fragility fractures of the pelvis (FFP) by Rommens and Hofmann differentiating isolated anterior or posterior pelvic injuries as well as a combinations of these including the degree of displacement and hence the resulting instability [13]. Isolated injuries of the anterior pelvic ring were classified as FFP type I. FFP type IIa represent non-displaced isolated fractures (unilateral or bilateral) of the sacrum (Fig. 2). FFP Type IIb and IIc are characterized by a non-displaced lesion of the posterior pelvic ring in combination with an anterior pelvic ring instability. In FFP type IIb injuries, there is a unilateral crush-zone in the sacral ala (Fig. 3), whereas in FFP type IIc injuries a complete non-displaced fracture of the ventral and dorsal sacral cortex is found (Figs. 4, 5). FFP type IIIc exhibit a higher degree of instability presenting a complete unilateral sacral disruption and a complete fracture of the anterior pelvic ring with some degree of displacement. A bilateral sacral fracture connected with a transverse fracture line is classified as FFP type IVb (Fig. 6), functionally being a highly unstable spinopelvic dissociation. A combination of bilateral posterior pelvic disruption including a sacral fracture is classified as FFP type IVc [13].

Typical sacral fracture patterns were described in a series of 85 FFS without bony pathology other than osteoporosis [40]. “H”-type fractures were described in 61 %, 12 % consisted of bilateral vertical fractures in the sacral alae, whereas in 19 % a unilateral vertical fracture line in the sacral ala was present. Half of these unilateral fractures (48 %) were accompanied by a hip pathology such as a hip arthorplasty, an avascular necrosis of the femoral head, or severe degenerative changes. In contrast, a hip pathology was discovered in only 8 % of cases with bilateral fractures [40].

A biomechanical model using finite element analysis showed that in a stance and walking model the highest stress was situated in the sacral ala, corresponding to the region where fractures in the osteoporotic sacrum are found. Simulating bilateral fractures in the sacral alae, maximal stress was noted horizontally connecting the vertical lines corresponding to the “H”-pattern seen in FFS [40].

## Therapy

**Fig. 7:**
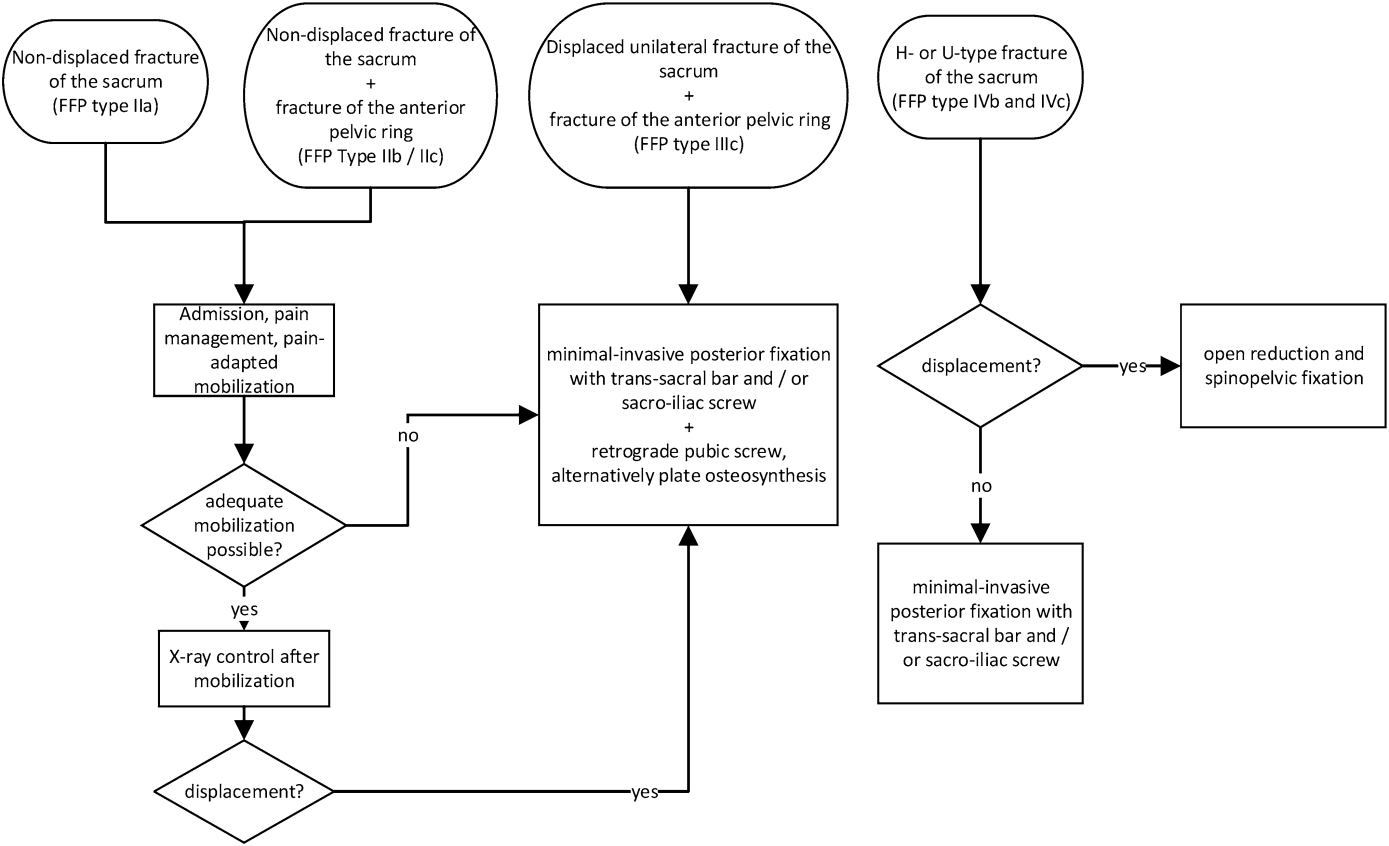
Therapeutic algorithm

The treatment of FFS must be individually adapted to the patient’s expectancy, his pre-traumatic level of mobility, the comorbidities, the duration of pain, and the fracture morphology. Non-displaced fractures of the sacrum with or without a combined anterior pelvic fracture (corresponding to FFP types IIa, IIb, or IIc of the Rommens and Hofmann classification [13]) are primarily treated non-surgically. However, some patients do suffer prolonged pain and require a more invasive treatment. In patients with displaced fractures (FFP type IIIc), we advocate a primarily minimal-invasive operative treatment. Patients with displaced bilateral sacral fractures (FFP types IVb and IVc) are treated surgically, as this spinopelvic dissociation harbors a high risk for fracture progression or displacement (Fig. 7).

## Conservative treatment

Conservative management is the primary approach for isolated non-displaced sacral fractures with or without an additional non-displaced fracture of the anterior pelvic ring [13] (Fig. 3). As these patients often suffer severe pain at mobilization, they are admitted to the ward and bed rest is advocated initially. Pain medication is used according to the WHO analgesic ladder with respecting the contraindications and the patient’s comorbidities. Mobilization and weight bearing as tolerated is started soon with the assistance of physiotherapists. The patient should not be forced at mobilization as this may increase the risk of fracture progression or displacement [13]. Early mobilization is important to prevent immobility-associated complications [48]. As long as patients are not properly mobilized, a prophylaxis of deep venous thrombosis according to local guidelines is applied.

After some days of mobilization, further fracture displacement is excluded by conventional radiographs. In case of persisting immobilization or pain, as well as fracture displacement, operative stabilization has to be taken into account.

## Operative treatment

We consider a primary surgical approach to be indicated in initially displaced fractures as in FFP type IIIc. Stabilization of the posterior pelvic ring leads to faster pain relief and mobilization. However, there still is no clinical evidence from larger case series or prospective investigations. As FFS often occur in elderly with multiple comorbidities, minimal-invasive techniques are to be favored taking the perioperative risks into account. Further, we recommend surgical treatment for patients with a non-displaced FFS which suffer ongoing immobilizing pain while treated conservatively.

In FFS combined with a fracture of the anterior pelvic ring, we recommend stabilization of both, the posterior and the anterior pelvic ring. When feasible, this is carried out in a minimal-invasive way [49, 50] (Fig. 4).

**Fig. 8:**
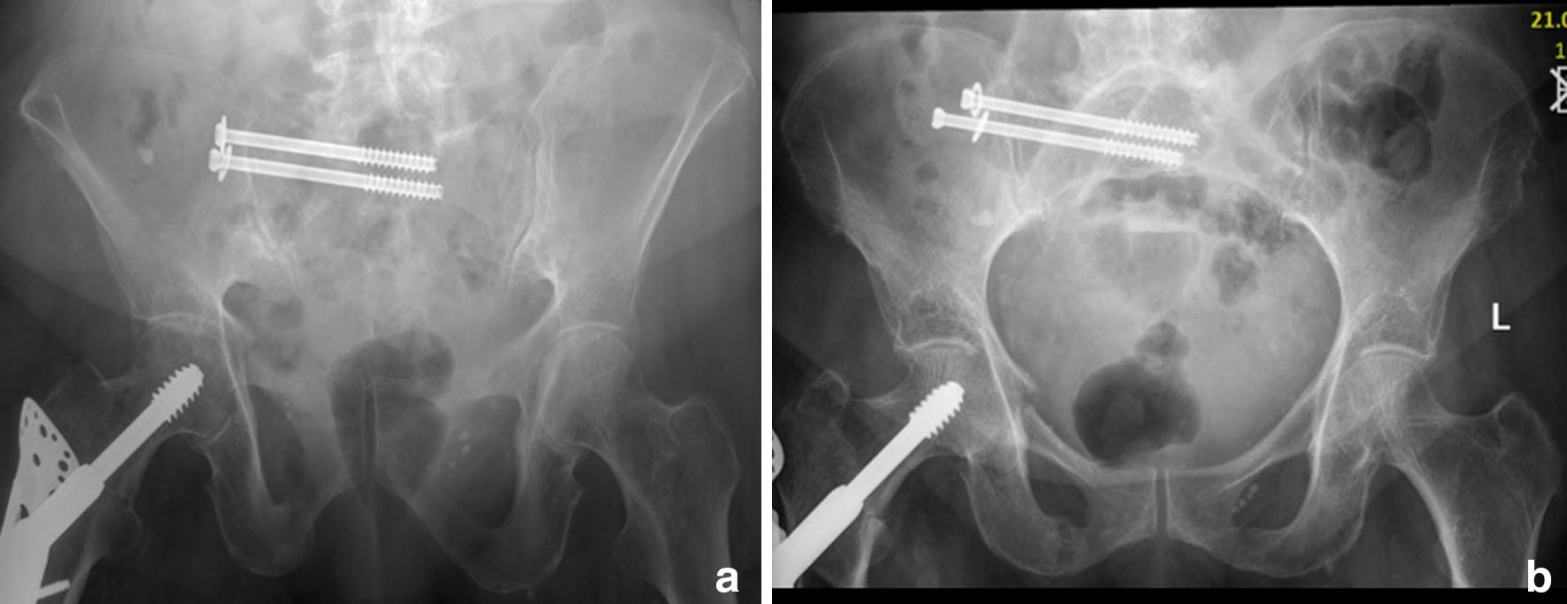
Backing out of SI-screw one month postoperatively in a 87-year-old female while only the posterior pelvic ring was fixed; however, mobilization was not painful

Fractures of the sacrum usually are stabilized with minimal-invasive sacro-iliac (SI) screws. These screws are inserted percutaneously with the patient either in prone or in supine position, crossing both, the SI-joint and the fractured area. They anchor in the vertebral body of S1 or S2 [51]. Insertion of SI-screws requires a thorough knowledge of the radiological anatomy of the pelvis and a meticulous preoperative planning. The highly variable anatomy of the upper sacrum may render placement of implants difficult due to the limiting space of safe corridors [52–54]. Respecting the individual anatomy, percutaneous implant positioning carries only a low risk of complications [55]. In this context, screw malpositioning was reported to occur in 1.8 % with an overall revision rate of 2 % [56]. However, screw loosening was reported in osteoporotic bone [57–59] (Fig. 8). A recent study of patients with an average age of 77 years treated with SI-screws demonstrated, as far as follow-up data was available, backing out of screws in 14 %, development of a contralateral sacral fracture in unilateral surgically treated sacral fractures in 8 %, and development of non-union in 9 % [59]. A so-called “alar void” located in the sacral ala was found as a zone of decreased bone mass compared to the vertebral body S1 [52, 60–62]. The screw purchase depends on the insertion depth and is better in the vertebral body than in the sacral ala depending on the local bone mineral density [63, 64]. In spine surgery, a better pullout resistance has been achieved using perforated pedicle screws which were augmented with PMMA (polymethylmethacrylate) cement [65]. This concept has been adopted to the sacrum by augmenting SI-screws, thereby applying PMMA-cement through perforated SI-screws or by insertion of the screw after cement application [66–68].

We prefer using trans-sacral implants to overcome the shortcomings of decreased bone mass and hence weaker screw anchorage in the sacrum [49, 50, 69, 70] (Figs. 2, 4, 5, 6). These implants traverse the sacrum on level S1 or S2, entering the iliac bone on one side, perforating the SI-joint, passing through the vertebral body to the contralateral side of the sacrum, and exiting there the iliac bone after crossing the SI-joint [52]. They are inserted through safe pathways called trans-sacral corridors, varying considerably in their size due to the highly variable anatomy of the upper sacrum [52, 54]. Considering this fact, a thorough preoperative planning is imperative because dysplastic morphology may render trans-sacral implant positioning at level S1 impossible. Alternatively, level S2 offers more consistent space to insert such an implant [52, 54, 71]. Biomechanically, the stability of trans-sacral implants depends on the compression forces applied to the cortices of the iliac bones and not on screw purchase in the weaker trabecular bone of the sacrum. Higher load to failure and less displacement were demonstrated in a biomechanical study using a locked trans-sacral implant along with a SI-screw compared to two SI-screws in an osteoporotic model of a vertical shear pelvic injury [72]. Significant compression forces can be achieved to the vertical fracture lines by tightening the nuts of the threaded trans-sacral rod. Thereby, an additional SI-screw can help reducing toggling and rotation of pelvic bone around the axis of the rod in the plane of the fracture (Figs. 2, 4, 6).

Displaced fractures of the sacrum (FFP type IVb) which represent a functional spinopelvic dissociation may require open reduction and spinopelvic fixation to reconstruct a connection between the pelvis and lumbar spine. This construct can be combined with an additional SI-screw or a trans-sacral bar to create a triangular osteosynthesis [73–75]. Another alternative treatment is the plate osteosynthesis of the posterior pelvis [76, 77] although we doubt the compressive force on the posterior pelvic ring exhibited by these constructs.

## Sacroplasty

In recent years, a minimal invasive technique for augmentation of sacral fractures with PMMA cement, the so called sacroplasty, became popular [33, 78]. This technique aims at early pain relief and faster mobilization. PMMA is injected into the sacral ala where the fracture is typically located [40] using a longitudinal or a short-axis approach [78]. A reduction of micromotion at the fracture site was shown in finite element analysis [33, 79] and in a cadaver test setup [80]. However, these results are somewhat contradictory to the results of another study showing no difference in strength and stiffness restoration after sacroplasty compared to the control group [81]. Clinically, a significant pain relief was found in patients treated with sacroplasty changing VAS (visual analogue scale) from 9.2 ± 1.1 points before to 1.9 ± 1.7 after sacroplasty [82] with patient’s mobility increasing significantly [83]. Leakage of PMMA cement is a major possible complication with this procedure. This was described to occur in one-third of cases; thereby PMMA cement leaked into the venous plexus, into the fracture gap, into the neuroforamina, and into disc space L5/S1 [84]. Surgical cement removal was necessary in a described case due to radiculopathy [85]. In kypho- and vetebroplasty, cement augmentation is used to counteract the vertical compression forces acting on vertebral bodies with horizontally orientated fracture lines. In the sacrum, however, the same axial loading creates shear forces along the vertically orientated fracture lines, which cannot be controlled by cement augmentation. Therefore, the beneficial biomechanical effect of sacroplasty seems questionable. Furthermore, cement injected into the fracture gap may hinder fracture healing [49, 86, 87].

## Osteoporosis and pharmacological treatment

It is of outstanding importance recognizing the osteoporotic nature of FFS and initiating an osteoporosis-workup and/or therapy. This is demonstrated by a study in elderly women suffering from a distal radius fracture, another typical fragility fracture, which thereafter underwent a diagnostic osteoporosis workup in only one-fourth of the patients and medical treatment was initiated in only 2 % [88]. Hence, the orthopedic surgeon dealing with patients suffering a FFS does and should play an important role in the initiation of osteoporosis workup and anti-osteoporosis treatment [89]. This is highlighted by the fact that fractures of the pelvis in elderly women were associated with a low BMD in the femoral neck and did pose a risk for the future occurrence of major osteoporotic fractures [90] (Fig. 5). Implementing a program of diagnostic workup and therapy initiation showed a decrease in future hip fracture rate of 31–54 % [91]. Diagnosis of osteoporosis is made using dual X-ray absorptiometry to determine the bone mineral density in the spine and the proximal femur; further, individual fracture risk is calculated by FRAX risk assessment tool. Laboratory testing is used to exclude secondary osteoporosis. The management of patients with osteoporosis is multi-modal, consisting of life style changes, fall prevention, vitamin D, and calcium supplementation as well as administration of antiresorptive drugs [62]. To accelerate bone healing, daily subcutaneous injection of parathyroid hormone (PTH) can be used as anabolic agent [92]. In osteoporotic pelvic fractures, this has been shown to lead to a faster fracture healing and less pain with a better functional outcome after 3 months [93]. However, the supplementary treatment of osteoporotic fractures with PTH is not accepted on a regular base and not supported yet by regional guidelines.

## Clinical outcome and complications

Fragility fractures of the pelvis always produce some degree of instability and may progress to fractures with increased instability (such as widening of fracture lines or secondary fractures; Fig. 5) when patients are forced to mobilize with full weight bearing [57, 94, 95]. Even after unilateral dorsal fracture fixation a progression from a uni- to a bilateral fracture has been reported [59]. Such increased instability may lead to longstanding courses of pain at mobilization and finally to bedridden patients. In patients treated non-surgically, the time to improvement of symptoms and full mobilization varies from 4 weeks to 3.3 months [21, 37, 96]. A complete resolution of pain and regain of independence was evident after 9 months in only 85 % [36]. Data concerning required time of bed rest with conservative treatment vary widely in the literature between 12 days and 8 weeks [25, 36, 97]. Immobilization, particularly in the elderly, leads to a high number of complications such as deep venous thrombosis, pulmonary embolism, decline of muscle strength, risk of pneumonia, pressure ulcers, or psychological changes [48], occurring in 20–52 % of patients suffering from a FFP [98–100]. The mean duration of hospital admission was reported to be 10–45 days [23, 99, 100] with significant longer stays in patients with a combined anterior and posterior pelvic ring injury [23]. Thereby, the early in-hospital mortality rate was 3–10 % [8, 23, 100, 101]. The high impact of FFP on the survival is evident considering the 1-year mortality of 11–19 % [8, 97, 101–103]. Patients with a FFP aged more than 90 years even showed a 1-year mortality of 39 % [102]. The overall 5-year mortality reached 54 %, increasing with age and dementia [8]; after 10 years the overall mortality rate reached 94 % which was statistically significantly higher than observed in an age-matched population [100]. In addition to the high mortality rate, the functional status also decreases after such an injury. One year after the fracture, only 16 % of the patients were able to mobilize without walking aids and only 18 % were able to live independently [101], half of patients lost their pre-traumatic autonomy [99].

Rare complications of FFP include massive hemorrhage [104] due to injury of the inferior epigastric artery [105–107], an avulsion of the corona mortis [108], or an injury to the obturator, the pudendal, or the internal iliac artery [107]. Bleeding after an isolated FFS was described due to an injury of the superior gluteal artery [108]. The occurrence of an infected hematoma of the psoas muscle as consequence of a FFS was reported [109]. Further, an intrapelvic abscess formation was described after a displaced fracture of the pubic rami due to a bladder puncture [110, 111].

Neurological damage was described to occur in 2.8 % of patients with FFS, e.g. sphincter dysfunction or root compression syndromes [25]; however, a cauda equina syndrome also can be caused by an expanding intraspinal hematoma [112].

## Conclusions

Elderly with low back pain, especially if a low-energy trauma occurred, should raise the suspicion of suffering from a FFS. Up to date, the incidence of FFS is frequently underestimated and often diagnosed with delay. Although conventional X-ray is the primary tool in the diagnostics of FFS and FFP, further diagnostics using CT or MRI should be undertaken to exclude a fracture of the posterior pelvic ring and in case of prolonged pain, to detect occult fractures. The management of FFS depends upon the fracture characteristics, the patient’s comorbidities, and their symptoms. Conservative treatment is initiated in non-displaced fractures; however, more invasive methods are considered in case of inadequate mobilization or persistence of pain. As displaced fractures are not stable, they are treated with minimal-invasive fracture fixation dorsally (preferably with trans-sacral bar) and anterior fixation if an anterior pelvic lesion is present. H- or U-type fracture patterns functionally represent a spinopelvic dissociation; they are unstable and should be fixed in a minimal invasive way in cases with no or only slight displacement. However, if gross displacement is present, a spinopelvic stabilization is recommended. The treating physician should keep in mind that FFS and FFP are associated with osteoporosis and initiate a workup and treatment to prevent future fragility fractures at other sites. FFS have a high impact on the patient’s health with an increase in morbidity and mortality; further, patients frequently experience loss of their autonomy.

## Abbreviations

FFS: Fragility fracture of the sacrum
FFP: Fragility fracture of the pelvis
SIF: Sacral insufficiency fracture
MRI: Magnet resonance imaging
PMMA: Polymethylmethacrylate
PTH: Parathyroid hormone
CT: Computed tomography
HU: Hounsfield units
